# PRMT5-dependent transcriptional repression of c-Myc target genes promotes gastric cancer progression

**DOI:** 10.7150/thno.42047

**Published:** 2020-03-15

**Authors:** Ming Liu, Bing Yao, Tao Gui, Chan Guo, Xiaobin Wu, Jiahuang Li, Lingling Ma, Yexuan Deng, Peipei Xu, Ying Wang, Dongjun Yang, Qixiang Li, Xiangwei Zeng, Xinyu Li, Ruifeng Hu, Jingru Ge, Zenong Yu, Yugen Chen, Bing Chen, Junyi Ju, Quan Zhao

**Affiliations:** 1 The State Key Laboratory of Pharmaceutical Biotechnology, Department of Hematology, the Affiliated Drum Tower Hospital of Nanjing University Medical School, China-Australia Institute of Translational Medicine, School of Life Sciences, Nanjing University, Nanjing, China; 2 Department of Medical Genetics, Nanjing Medical University, Nanjing, China; 3 The Affiliated Hospital of Nanjing University of Chinese Medicine, Nanjing, China

**Keywords:** PRMT5, c-Myc, gastric cancer, histone arginine methylation, tumorigenesis

## Abstract

The proto-oncogene c-Myc regulates multiple biological processes mainly through selectively activating gene expression. However, the mechanisms underlying c-Myc-mediated gene repression in the context of cancer remain less clear. This study aimed to clarify the role of PRMT5 in the transcriptional repression of c-Myc target genes in gastric cancer.

**Methods**: Immunohistochemistry was used to evaluate the expression of PRMT5, c-Myc and target genes in gastric cancer patients. PRMT5 and c-Myc interaction was assessed by immunofluorescence, co-immunoprecipitation and GST pull-down assays. Bioinformatics analysis, immunoblotting, real-time PCR, chromatin immunoprecipitation, and rescue experiments were used to evaluate the mechanism.

**Results**: We found that c-Myc directly interacts with protein arginine methyltransferase 5 (PRMT5) to transcriptionally repress the expression of a cohort of genes, including PTEN, CDKN2C (p18^INK4C^), CDKN1A (p21^CIP1/WAF1^), CDKN1C (p57^KIP2^) and p63, to promote gastric cancer cell growth. Specifically, we found that PRMT5 was required to promote gastric cancer cell growth *in vitro* and *in vivo*, and for transcriptional repression of this cohort of genes, which was dependent on its methyltransferase activity. Consistently, the promoters of this gene cohort were enriched for both PRMT5-mediated symmetric di-methylation of histone H4 on Arg 3 (H4R3me2s) and c-Myc, and c-Myc depletion also upregulated their expression. H4R3me2s also colocalized with the c-Myc-binding E-box motif (CANNTG) on these genes. We show that PRMT5 directly binds to c-Myc, and this binding is required for transcriptional repression of the target genes. Both c-Myc and PRMT5 expression levels were upregulated in primary human gastric cancer tissues, and their expression levels inversely correlated with clinical outcomes.

**Conclusions**: Taken together, our study reveals a novel mechanism by which PRMT5-dependent transcriptional repression of c-Myc target genes is required for gastric cancer progression, and provides a potential new strategy for therapeutic targeting of gastric cancer.

## Introduction

Despite advances in early diagnosis, surgical resection, and adjuvant chemotherapy, gastric cancer is still one of the most aggressive malignancies with high morbidity and mortality 1. Although several candidate therapeutic targets have been identified, the clinical efficacy related to these targets has been disappointing 2, 3. Therefore, there is an urgent need to better understand the pathogenesis of gastric cancer, which could identify novel related biomolecules for new diagnostic, therapeutic, and preventive approaches for this deadly disease.

The proto-oncogene c-Myc is a basic helix-loop- helix/leucine zipper (bHLH-Zip) transcriptional regulator that controls multiple biological processes, including cell growth and differentiation, as well as tumor initiation and progression 4-8. c-Myc is documented as the most frequently amplified oncogene, and dysregulation of c-Myc correlates with tumor aggression and poor clinical outcome in the majority of malignancies. Key to the tumor-promoting functions of c-Myc as a transcription factor is its capacity to bind specific DNA elements within the regulatory regions of its target genes. c-Myc preferentially binds the canonical “E-box” motif (CACGTG or its variants CANNTG) within the proximal promoter or enhancer regions of target genes through dimerizing with its partner MAX 6, 9. Unlike the majority of transcription factors, c-Myc promotes transcriptional amplification generating elevated levels of transcripts from existing gene expression rather than stimulating transcription initiation. In this regard, Myc targets are normally dictated by opening chromatin accessibility, which allows Myc to bind target genes and cooperate with other gene regulators to selectively activate gene expression 10-12. Myc has also been shown to repress transcription of a large number of genes mainly by interacting with transcription factors, MIZ-1, SP1/SP3, and NF-YB/NF-YC repressing their activation 6. It has also been shown to mediate transcriptional repression by activating miRNAs, and recruiting histone deacetylases and DNA methyltransferases 6, 13. However, there is a more limited understanding of the role of c-Myc in transcriptional repression particularly within the context of specific cancer types.

Arginine methylation of histone tails is a post-translational modifications (PTMs) that has been linked to both transcription activation and repression 14. Protein arginine methyltransferases (PRMTs) catalyze mono- and di-methylation of arginine residues in the presence of a methyl donor (S-adenosyl methionine, SAM). Di-methylation of the guanidine group of arginine residues generates two types of di-methylarginine, symmetrical (SDMA) and asymmetrical (ADMA) 15, 16. PRMT5 is the major symmetric arginine methyltransferase in mammalian cells, which might be a novel therapeutic target molecule for human tumors 17, 18. It deposits symmetric di-methylation on histone substrates (H4R3, H3R2, H3R8 and H2AR3) as well as on other cellular proteins 19, 20. PRMT5-mediated arginine methylation modulates a variety of cellular processes including cell growth 20, 21, metastasis 22, 23, ribosome biogenesis 24, cellular differentiation 25, 26, gene transcription 27-29, germ cell specification 30, alternative splicing 31, 32 and Golgi apparatus formation 33. Although ChIP-seq analysis revealed that H4R3me2s is associated with global repressive genes 34, how PRMT5 is recruited to and functions at its target genes remains unknown.

Dysregulation of epigenetic mechanisms is a distinct feature of cancer. Recent reports associate elevated levels of PRMT5 with several human diseases, especially in cancer, including lung cancer, breast cancer, leukemia, lymphoma, gastric cancer, and colorectal cancer 16, 19. PRMT5 loss in gastric cancer cells has also been shown to inhibit tumorigenesis *in vitro* and in xenograft models, which has been suggested to occur via epigenetic silencing of the tumor suppressor IRX1 although the role of H4R3me2s was unclear 23.

In the present study, we provide evidence of a direct and functional link between PRMT5-mediated H4R3me2s and c-Myc in gastric cancer. The PRMT5-mediated H4R3me2s mark is enriched on c-Myc-binding CANNTG E-box elements and acts together with c-Myc to selectively repress expression of a cohort of largely cell cycle-related genes, including PTEN, CDKN2C (p18^INK4C^), CDKN1A (p21^CIP1/WAF1^), CDKN1C (p57^KIP2^) and p63, to promote gastric cancer cell growth. Our results thus unravel novel mechanisms of c-Myc-mediated transcriptional repression and of PRMT5 recruitment and function within the context of gastric cancer, revealing a new potential strategy for targeted therapy.

## Materials and Methods

### Cell lines

Human gastric cancer cell lines BGC823 and SGC7901 were obtained from the Cell Bank of the Chinese Academy of Sciences (Shanghai, China) and cultured in RPMI-1640 medium contained 10% fetal bovine serum (FBS, Gibco) and 1% penicillin- streptomycin (Beyotime Biotechnology) in a humidified atmosphere of 5% CO_2_ at 37°C. The human gastric cancer cell lines were recently authenticated by Genetic Testing Biotechnology Corporation (Suzhou, China) using short tandem repeat (STR) profiling. All lines were found to be negative for mycoplasma contamination.

### Tissue microarrays and immunohistochemical staining

Tissue microarrays (TMAs) with clinical pathological data were provided by Shanghai Biochip Co., Ltd. (Shanghai, China). The arrays contained gastric cancer tissue samples and matched normal tissues adjacent to the tumor. The paraffin-embedded tissues were deparaffinized, rehydrated, and then subjected to antigen retrieval. The tissues slides were incubated with PRMT5, Ki-67, c-Myc, PTEN and p57 antibodies overnight at 4°C. Subsequently, the slides were incubated with horseradish peroxidase (HRP)- conjugated secondary antibody. All gastric cancer tissue sections were reviewed by two experienced pathologists, and staining of target proteins in the tissue was scored independently by two pathologists blinded to the clinical data adopting the semiquantitative immunoreactive score (IRS) system 35, 36.

### siRNA, shRNA and infection

Specific siRNAs targeting PRMT5 and c-Myc were synthesized by GenePharma (Shanghai, China). Cells were transiently transfected with siRNA duplexes to a final concentration of 100 nM using Lipofectamine 3000 (Invitrogen). siRNA sequences targeting PRMT5 were siRNA-1: 5'-GCACCAGUCUGUUCUGCUA-3'; siRNA-2: 5'-GGCGAUGCAGCAAUUCCAA-3'; siRNA-3: 5'-GGACCUGAGAGAUGAUAUA-3'. siRNA sequences targeting c-Myc were siRNA-1: 5'-CGAUGUUGUUUCUGUGGAA-3'; siRNA-2: 5'-CCAAGGUAGUUAUCCUUAA-3'. ShRNA lentivirus were prepared by the co-transfection of 293T cells with pLKO.1 and packaging plasmids pMD2G and psPAX2. 48 h after transfection, the viral supernatants were collected. The BGC823 or SGC7901 cells were incubated with viral supernatants in the presence of polybrene. The positive cells were selected with 1μg/mL puromycin. The target sequences were PRMT5 shRNA-1: 5'-CCCATCCTCTTCCCTATTAAG-3'; PRMT5 shRNA-2: 5'-GCCCAGTTTGAGATGCCTTAT-3'. PTEN shRNA were 5'-CTAGAACTTATCAAACCCTTT-3'. p18 shRNA were 5'-CTATGGGAGGAATGAGGTTGT-3'. p21 shRNA were 5'-GACAGATTTCTACCACTCCAA-3'. p57 shRNA were 5'-CCACGCACTAGCTCGGTTATT-3'. p63 shRNA were 5'-GCCACATCAAACCTTTGAGTA-3'. Scramble sequence was used as negative controls.

### CCK-8, EdU and colony-formation assays

For the CCK-8 assay, cells were seeded in 96-well plates in RPMI-1640 medium containing 10% FBS at equal density of 2×10^3^ cells/well. After incubation with CCK-8 reagent (a311-01, Vazyme Biotech) for 1 h at 37°C, the absorbance at 450 nm was determined by a spectrophotometer. EdU incorporation assay was performed using the EdU DNA Cell Proliferation Kit (C10310-3, Ribobio). In brief, cells were incubated with 50 μM EdU for 16 h at 37°C, fixed with 4% paraformaldehyde for 30 min and permeabilized with 0.3% Triton X-100 for 20 min. After washing with PBS, the cells were incubated in Apollo staining solution, and then stained with DAPI. For the colony-formation assay, cells were seeded sparsely in 6-well plates with normal medium at a density of 1000 cells/well. Two weeks later, colonies were fixed with methanol and stained with 0.5% (w/v) crystal violet solution at room temperature. After washing with distilled water, the colonies were counted visually.

### Recombinant protein expression and purification

pGEX-6p-1 plasmids encoding GST, GST-PRMT5 (wild-type), GST-PRMT5 F1, F2, F3, GST-PRMT5 488-490Δ, GST-PRMT5 R488A and GST-PRMT5 K490A, and pET28a plasmid encoding His-tagged c-Myc were transformed into *E. coli* BL21, and cultured with IPTG at 16°C for 12 h until the optical density (OD600) reached 0.5 ~ 0.6. BL21 cells were collected, sonicated in cold PBS and purified with Glutathione S-transferase (GenScript, L00206) beads or Nickel-nitrilotriacetic acid (GenScript, L00250) beads according to the users' manual.

### Protein extraction and immunoblotting

Cells were trypsinized, washed with phosphate- buffered saline (PBS) and lysed in Cell lysis buffer (20 mM Tris at pH 7.5, 150 mM NaCl, 1% Triton X-100, Sodium pyrophosphate, β-glycerophosphate, EDTA, Na_3_VO_4_, leupeptin). Equal amounts of protein were separated by 8 ~ 15% SDS-PAGE and transferred to polyvinylidene difluoride (PVDF) membranes (Roche). The membranes were blocked for 1 ~ 2 h at room temperature in PBST with 5% (w/v) non-fat milk and incubated overnight at 4°C with primary antibodies. After incubation with secondary antibodies, proteins were visualized with the ECL detection system (Thermo Fisher Scientific). The following antibodies were used: PRMT5 (P0493, Sigma), c-Myc (ab32072, ab56, Abcam), GAPDH (M171-3, MBL), PTEN (ab32199, Abcam), p21 (#2947, CST), p63 (ab124762, Abcam), p18 (ab192239, Abcam), p57 (ab75974, Abcam), HSP70 (#4873, CST), H4R3me2s (ab5823, Abcam) and Histone H4 (16047-1-AP, Proteintech).

### Quantitative real-time PCR

Total RNA was isolated using TRIzol reagent (Invitrogen) according to the manufacturer's instructions. The cDNA was synthesized using a HiScript Q RT SuperMix kit (R123-01, Vazyme Biotech). Quantitative real-time PCR analysis was performed using qPCR SYBR Green Master mix (Q311-02, Vazyme Biotech). The qPCR plates were denatured for 5 min at 95°C, and then subjected to 40 cycles of 20 s at 95°C, 15 s at 60°C and 25 s at 72°C in a CFX96 qPCR detection system (Bio-Rad). Relative mRNA expressions were calculated using the 2^-ΔΔCt^ method. GAPDH was employed as the endogenous control. The primer sequences are listed in SI Appendix Table S1.

### Immunofluorescence

Cells were cultured on glass coverslips, fixed with 4% paraformaldehyde (Sigma) for 30 min and permeabilized by 0.3%Triton X-100 for 30 min at room temperature. After blocking with 5% goat serum in PBS, cells were incubated with primary antibodies for 60 min and secondary antibodies for 45 min in a dark and humid chamber, respectively. Then, cell were washed with PBS and the nuclei stained with 4, 6, diarnidino-2-phenylindole (DAPI) for 5 ~ 10 min. Immunofluorescence images were captured under a fluorescence microscope (BX53, Olympus).

### Chromatin immunoprecipitation (ChIP)

Cells were cultured in RPMI-1640 with 10% FBS, and then cross-linked with 1% formaldehyde at room temperature. 10 ~ 15 min later, the reaction was quenched with Glycine for 10 min at a final concentration of 125 mM. Subsequently, the chromatin was sonicated to produce DNA fragments (200 bp ~ 500 bp). 100 μg chromatin was incubated overnight with rotation at 4°C with primary antibodies or IgG (2 μg) followed by 1 h incubation with protein A sepharose beads. After washing with low-salt buffer and high-salt buffer, the beads were incubated with elution buffer and proteinase K at 65°C overnight to immunoprecipitate DNA. Total DNA fragments were isolated by phenol/chloroform extraction and ethanol precipitation. After isolation, the DNA was diluted in water and subjected to real-time PCR analysis. The primers used for the analysis are listed in Table S2.

### shRNA-resistant PRMT5 constructs

To overexpress PRMT5 or mutant in PRMT5-depleted BGC823 cells, PRMT5 (wild-type), PRMT5 Δ or PRMT5 R368A (enzymatically inactive), and PRMT5 K490A cDNAs were cloned into the eukaryotic expression pcDNA3.1 plasmid. The shRNA-resistant PRMT5 or mutant cDNAs were generated by introducing point mutations into the target site of PRMT5 shRNA-1. The target sequence 5'-CCCATCCTCTTCCCTATTAAG-3' was mutated to 5'-CCGATTTTGTTTCCCATAAAA-3'-. The amino acid sequence of protein did not change.

### *In vivo* xenograft growth assay

All animal care and handling procedures were performed in accordance with the National Institutes of Health Guide for the Care and Use of Laboratory Animals, and were approved by the Institutional Review Board of Nanjing University (Nanjing, China). BALB/c nude mice (female, 6 ~ 8 weeks) were purchased from the Model Animal Research Center of Nanjing University (Nanjing, China). Scr, sh_PRMT5- treated and sh_PRMT5 + sh_p57-treated BGC823 cells were suspended in PBS containing 10% Matrigel (BD Biosciences) and injected subcutaneously into the flanks of nude mice (1×107 cells per mouse). The sizes of tumors (Length and Width) were measured every 4 days and tumor volumes were calculated using the formula: Volume (cm3) = 0.5 × (Length) × (Width)^2^. 20 days later, all nude mice were sacrificed and tumors were excised, photographed and weighed.

### Motif analysis

Publicly available H4R3me2s ChIP-seq data sets from mouse embryonic stem cells were obtained from the GEO database (GSE37604). To identify DNA sequence motifs enriched in H4R3me2s regions, we used Hypergeometric Optimization of Motif EnRichment (HOMER) for the motif analysis. Reducing redundancy by clustering and merging motifs was performed as in a previous report 37.

### Statistical analysis

Statistical analysis was performed by Student's *t* test for comparing two groups using the GraphPad Prism software. Data are shown as mean ± SD. Differences in the mean values were considered to be significant at *P* < 0.05.

## Results

### PRMT5 is upregulated in gastric cancer and required for cell proliferation *in vitro*

**Figure 1:**
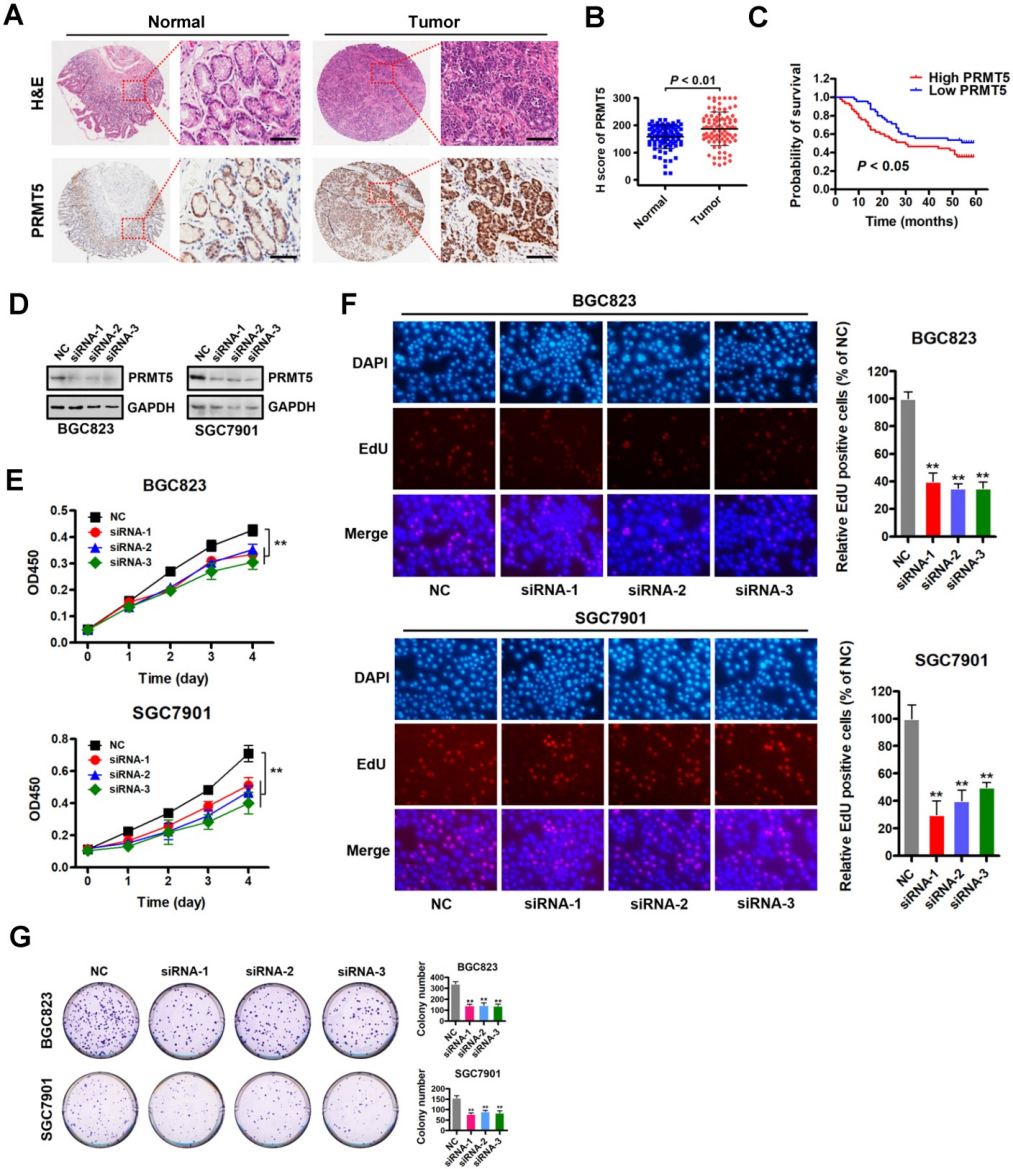
** PRMT5 is upregulated in gastric cancer and is required for cell proliferation *in vitro.* (A)** Representative images of H&E and immunohistochemical staining (IHC) of PRMT5 in gastric cancer (n = 90; right panel) or adjacent noncancerous (n = 90; left panel) tissues. The boxed areas in the left images are magnified in the right images. Scale bar: 50 µm. **(B)** IHC score of PRMT5 in gastric cancer (n = 90) and adjacent noncancerous (n = 90) tissues, *P* < 0.01. **(C)** Overall survival of High PRMT5 (n = 45) and Low PRMT5 (n = 45) gastric cancer patients was compared by Kaplan-Meier survival analysis, *P* < 0.05. **(D)** Protein levels of PRMT5 were examined by Western blot analysis in BGC823 and SGC7901 cells transfected with PRMT5 siRNAs or negative control (NC). GAPDH was served as internal controls. **(E)** Cell proliferation was assessed by CCK-8 assays at days 1, 2, 3 and 4 in BGC823 and SGC7901 cells transfected with PRMT5 siRNAs or NC. Data shown are mean ± SD (n = 3). **P < 0.01. **(F)** EdU incorporation was assessed in BGC823 and SGC7901 cells transfected with PRMT5 siRNAs or NC. Quantitative analysis of EdU positive cells (% of NC) is shown in the bar graph (right panels). Data shown are mean ± SD (n = 3). **P < 0.01. **(G)** Colony-formation was assessed in BGC823 and SGC7901 cells transfected with PRMT5 siRNAs or NC. Colony numbers were shown in the bar graph (right panels). Data shown are mean ± SD (n = 3). **P < 0.01.

To investigate the role of PRMT5 in gastric cancer, we first examined PRMT5 expression by immunohistochemistry using tissue arrays containing 90 pairs of gastric cancer and their matched non-tumorous tissues (Table S3). The summarized IHC data indicated that PRMT5 expression was significantly upregulated in gastric cancer tissues compared with matched adjacent normal tissues (Figure 1A-B). Notably, Kaplan-Meier survival analysis showed that high PRMT5 expression was linked with poor overall survival up to thirty months at least (Figure 1C). These results indicate that PRMT5 is upregulated in gastric cancer and suggest that high PRMT5 expression is associated with poor prognosis in gastric cancer patients.

Based on this PRMT5 expression analysis in gastric cancer samples, we reasoned that depleting PRMT5 would attenuate the malignancy of gastric cancer cells. Therefore, we designed three specific siRNAs targeting PRMT5, and their efficacy was verified by Western blot analysis in gastric cancer cells BGC823 and SGC7901 (Figure 1D). CCK-8 assays showed that knockdown of PRMT5 attenuated proliferation of BGC823 and SGC7901 cells compared to NC controls (Figure 1E). The results were further confirmed by EdU incorporation and colony- formation assays in both BGC823 and SGC7901 cells (Figure 1F-G). These results indicate that PRMT5 is required for gastric cancer cell proliferation *in vitro*.

### Identification of downstream targets of PRMT5 in gastric cancer cells

**Figure 2:**
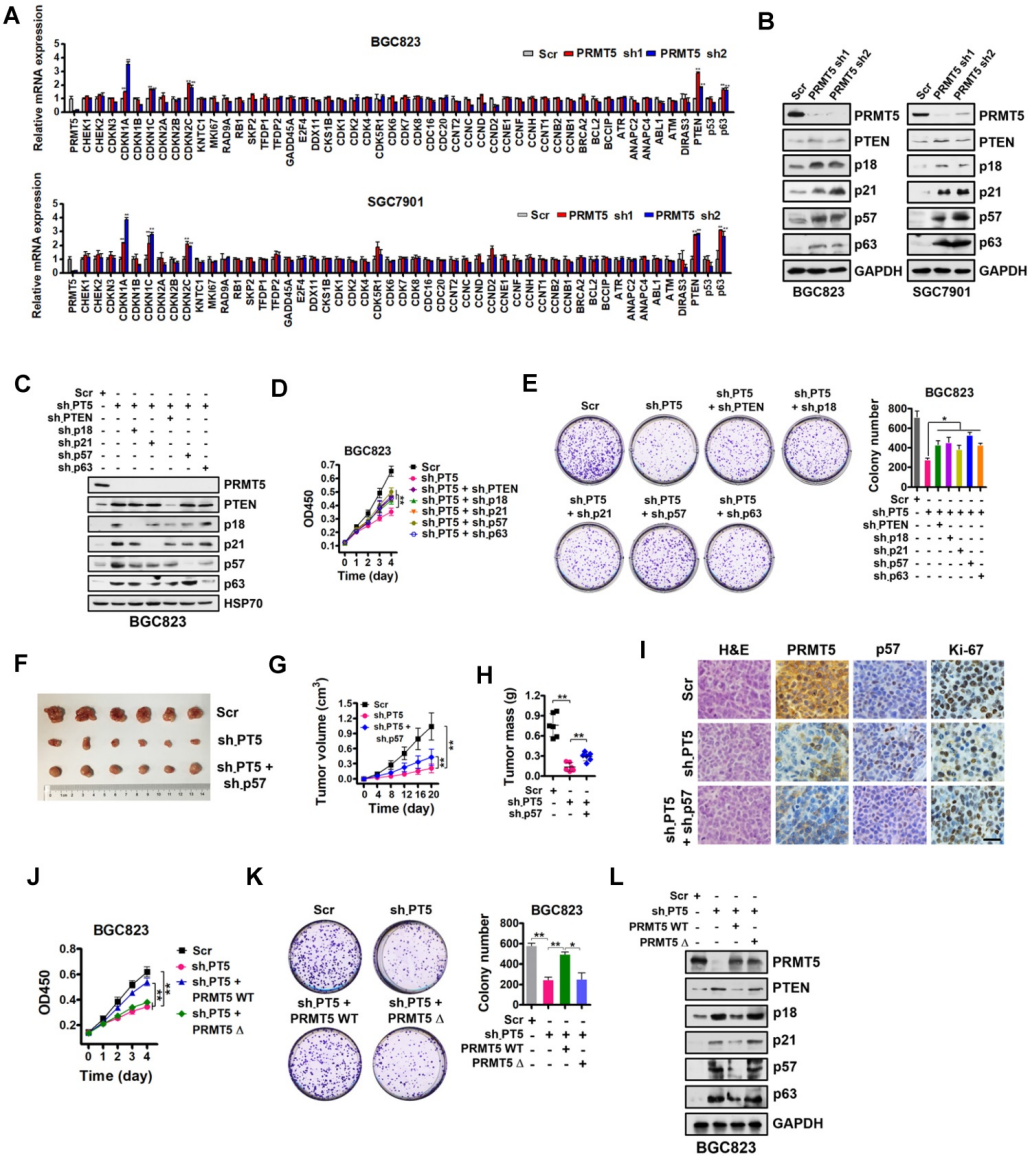
** Identification of downstream targets of PRMT5 in gastric cancer cells. (A)** Relative mRNA expression levels of a panel of key genes involved in cell proliferation and cell cycle regulation were analyzed by quantitative real-time PCR in scrambled control (Scr) or PRMT5-silenced (PRMT5 sh1/2) BGC823 and SGC7901 cells. GAPDH was used as an endogenous control. Data shown are mean ± SD (n = 3). **P < 0.01. **(B)** Immunoblots of PTEN, p18, p21, p57 and p63 protein levels in PRMT5-silenced BGC823 and SGC7901 cells. GAPDH was served as a loading control. **(C)** PTEN, p18, p21, p57 and p63 knockdown by shRNAs were verified by Western blot in PRMT5-silenced BGC823 cells. HSP70 was served as a loading control. **(D)** Cell proliferation was assessed by CCK-8 assay at days 1, 2, 3 and 4 in Scr, sh-PT5-treated, sh-PT5 + sh-PTEN-treated, sh-PT5 + sh-p18-treated, sh-PT5 + sh-p21-treated, sh-PT5 + sh-p57-treated or sh-PT5 + sh-p63-treated BGC823 cells. Data shown are mean ± SD (n = 3). **P < 0.01. **(E)** Colony-formation was determined in Scr, sh-PT5-treated, sh-PT5 + sh-PTEN-treated, sh-PT5 + sh-p18-treated, sh-PT5 + sh-p21-treated, sh-PT5 + sh-p57-treated or sh-PT5 + sh-p63-treated BGC823 cells. Colony numbers are shown in the bar graph (right panel). Data shown are mean ± SD (n = 3). *P < 0.05. **(F)** Representative images of Scr, sh-PT5-treated or sh-PT5 + sh-p57-treated BGC823 xenograft tumors at day 20. **(G)** Growth curves of Scr, sh-PT5-treated or sh-PT5 + sh-p57-treated BGC823 xenograft tumors. Data shown are mean ± SD (n = 6). **P < 0.01. **(H)** Average tumor weights of Scr, sh-PT5-treated or sh-PT5 + sh-p57-treated BGC823 xenografts (n = 6). **P < 0.01. **(I)** Representative images of H&E and IHC staining for PRMT5, p57 and Ki-67 expression from Scr, sh-PT5-treated or sh-PT5 + sh-p57-treated BGC823 xenografts. Scale bar: 20 µm. **(J)** Cell proliferation was assessed by CCK-8 assay at days 1, 2, 3 and 4 in Scr, sh-PT5-treated, sh-PT5 + PRMT5 WT-treated or sh-PT5 + PRMT5Δ (enzymatically inactive)-treated BGC823 cells. Data shown are mean ± SD (n = 3). **P < 0.01. **(K)** Colony-formation was assessed in Scr, sh-PT5-treated, sh-PT5 + PRMT5 WT-treated or sh-PT5 + PRMT5Δ (enzymatically inactive)-treated BGC823 cells. Colony numbers were shown in the bar graph (right panel). Data shown are mean ± SD (n = 3). *P < 0.05. **(L)** Immunoblots of PTEN, p18, p21, p57 and p63 protein in Scr, sh-PT5-treated, sh-PT5 + PRMT5 (wild type)-treated or sh-PT5 + PRMT5Δ (enzymatically inactive)-treated BGC823 cells. GAPDH served as a loading control.

In order to identify potential transcriptional targets of PRMT5 in gastric cancer, a comprehensive analysis was performed by utilizing a quantitative RT-PCR array to profile the targets of PRMT5 in BGC823 and SGC7901 cells. Relative gene expression changes of a spectrum of 51 key genes involved in proliferation and cell cycle regulation was explored, including CHEK1, CHEK2, CDKN3, CDKN1A (p21^CIP1/WAF1^), CDKN1B (p27^KIP1^), CDKN1C (p57^KIP2^), CDKN2A (p16^INK4^), CDKN2B (p15^INK4b^), CDKN2C (p18^INK4C^), KNTC1, MKI67, RAD9A, RB1, SKP2, TFDP1, TFDP2, GADD45A, E2F4, DDX11, CKS1B, CDK1, CDK2, CDK4, CDK5R1, CDK6, CDK7, CDK8, CDC16, CDC20, CCNT2, CCNC, CCND, CCND2, CCNE1, CCNF, CCNH, CCNT1, CCNB2, CCNB1, BRCA2, BCL2, BCCIP, ATR, ANAPC2, ANAPC4, ABL1, ATM, DIRAS3, PTEN, p53 and p63. Our findings revealed that five of these genes, specifically PTEN, CDKN2C (p18^INK4C^), CDKN1A (p21^CIP1/WAF1^), CDKN1C (p57^KIP2^) and p63, were significantly upregulated in PRMT5-depleted BGC823 and SGC7901 cells (Figure 2A). Western blot analysis showed that PTEN, CDKN2C (p18^INK4C^), CDKN1A (p21^CIP1/WAF1^), CDKN1C (p57^KIP2^) and p63 expression were consistently increased in PRMT5-knockdown cells, validating the quantitative RT-PCR results (Figure 2B).

To further confirm that PTEN, p18, p21, p57 and p63, which have been linked with cancer cell growth 38-42, are downstream targets of PRMT5, we designed shRNAs against PTEN, p18, p21, p57 and p63, and knocked down expression of these genes in PRMT5-depleted BGC823 cells. Knockdown efficiencies were assessed by Western blot analysis (Figure 2C). Then, we performed cell proliferation and colony-formation assays with these cells. We found that knockdown of PTEN, p18, p21, p57 or p63 partially restored defects in proliferation and colony formation in the PRMT5-depleted BGC823 cells (Figure 2D-E). To further support this, we used p57 knockdown as an example and went on to perform *in vivo* experiments with mouse xenograft models. Knockdown of p57 partially reversed the proliferation defect in PRMT5-depleted BGC823 cells *in vivo* (Figure 2F-I). Together, our results demonstrate that PRMT5 is important for gastric cancer cell growth potentially involving PTEN, p18, p21, p57 or p63 gene repression.

To examine whether the methyltransferase activity of PRMT5 is necessary for the repression of PTEN, p18, p21, p57 or p63 gene expression, we constructed a sh-PRMT5-insensitive PRMT5 mutant with a deletion of amino acids 365-369 in the SAM binding motif (PRMT5Δ, enzymatically inactive) by site-directed mutagenesis 43. We found that wildtype PRMT5 (PRMT5 WT) could rescue the defective cell proliferation and colony formation in PRMT5-depleted BGC823 cells, whereas PRMT5Δ had no effect (Figure 2J-K). Next, we found that restoration of wildtype PRMT5 expression could abolish the upregulation of PTEN, p18, p21, p57 and p63 in PRMT5-depleted BGC823 cells. In contrast, no change in the expression of PTEN or other targets was observed in PRMT5-depleted BGC823 cells when overexpressing the enzymatically inactive PRMT5Δ (deletion of amino acids 365-369; Figure 2L) or PRMT5 R368A (Figure S1) 44, 45. These results indicate that the methyltransferase activity of PRMT5 is essential for repressing gene expression of PTEN, p18, p21, p57 and p63 in gastric cancer cells.

### PRMT5-mediated H4R3me2s are enriched at the E-box motif

**Figure 3:**
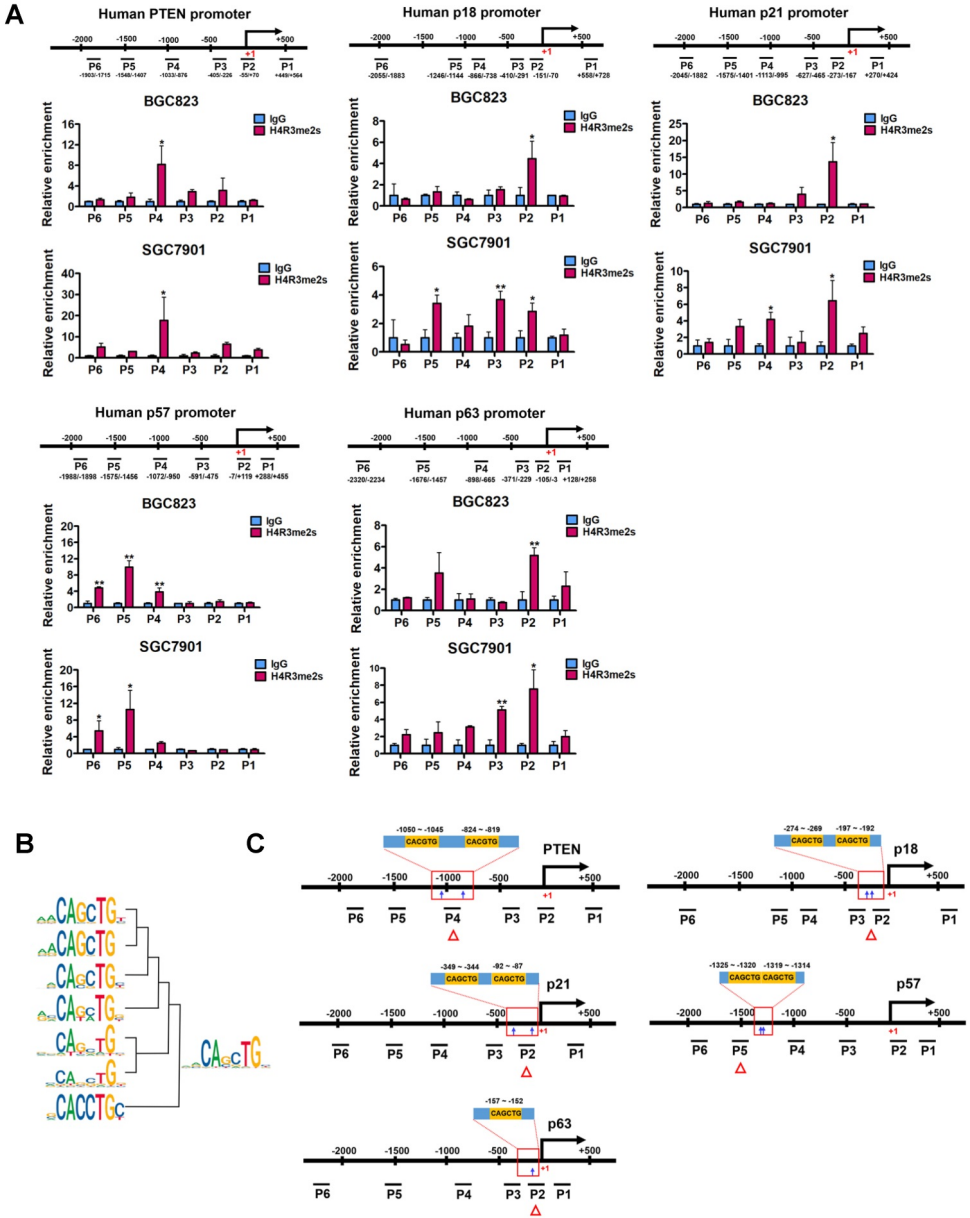
** PRMT5-mediated H4R3me2s is enriched at the E-box motif. (A)** PRMT5-mediated H4R3me2s modifications are enriched at the core promoter regions of PTEN (P1 ~ P6), p18 (P1 ~ P6), p21 (P1 ~ P6), p57 (P1 ~ P6) and p63 (P1 ~ P6) genes in BGC823 and SGC7901 cells by ChIP analysis. IgG was used as a negative control. Data shown are mean ± SD (n = 3). *P < 0.05, **P < 0.01. **(B)** Seven CANNTG motifs enriched in H4R3me2s regions (threshold *P* < 1e^-12^) were clustered and merged to a conserved sequence. **(C)** CACGTG or CAGCTG motif distribution in the H4R3me2s-enriched regions of PTEN, p18, p21, p57 and p63 promoters in BGC823 and SGC7901 cells.

In order to test whether PRMT5 directly regulates PTEN, p18, p21, p57 and p63 expression, we analyzed the enrichment of PRMT5-mediated H4R3me2s on the promoters of these candidate genes in BGC823 and SGC7901 cells using chromatin immunoprecipitation (ChIP) assays. Herein, we designed 4 to 6 pairs of walking primers across the core promoter regions of these candidate genes. We found that, in comparison with IgG controls, H4R3me2s was indeed enriched and distributed at the proximal promoter regions of PTEN, p18, p21, p57 and p63 genes in both BGC823 and SGC7901 cells (Figure 3A). These results suggest that PRMT5 can directly regulate PTEN, p18, p21, p57 and p63 gene expression via H4R3me2s.

To search for DNA motifs that were significantly enriched in H4R3me2s regions at a global level, we performed sequence motif analysis on publicly available H4R3me2s ChIP-Seq datasets from mouse embryonic stem cells (accession number GSE37604). A total of 16 highly enriched motifs were identified in increased H4R3me2s regions (threshold at *P* < 1×10^-12^, Table S4). Seven of these 16 motifs contained the CANNTG sequence that coincides with the evolutionarily conserved E-box sequence, which is a binding site of the basic helix-loop-helix (bHLH) superfamily of transcription factors. We clustered and merged these seven motifs to form a new motif, CAGCTG (Figure 3B), and found that this new motif exactly corresponds to an E-box variant element that c-Myc binds, suggesting it may also play a functional role in PRMT5-mediated transcriptional repression. Together, these results suggest that PRMT5-mediated H4R3me2s has a preferential neighboring DNA motif, CAGCTG, which might be associated with transcriptional repression.

Next, we verified that this coincidence occurs on PTEN, p18, p21, p57 and p63 genes. We scanned the core promoter regions of these genes for CANNTG motif distribution. CANNTG motifs were indeed observed in the H4R3me2s-enriched regions of PTEN, p18, p21, p57 and p63 core promoters (Figure 3C). We found two classical E-box elements (CACGTG) near the H4R3me2s-enriched regions on the PTEN promoter, whereas E-box variant elements (CAGCTG) were observed on the promoter regions of p18, p21, p57 and p63 genes near the H4R3me2s- enriched regions (Figure 3A and 3C). In contrast, we did not observe significant H4R3me2s enrichment on the promoter region of p15 (CDKN2B) gene which harbors a CAGCTG motif (Figure S2) 46. Of note, p15 expression was unaffected when PRMT5 was knocked down in BGC823 cells (Figure 2A). These results are consistent with the DNA motif sequence analysis of H4R3me2s-enriched regions and further confirmed that PRMT5-mediated H4R3me2s is enriched at the E-box or its variant motif at the promoters of PTEN, p18, p21, p57 and p63 genes.

### c-Myc is co-enriched with H4R3me2s at the PRMT5-targeted genes and represses their expression

**Figure 4:**
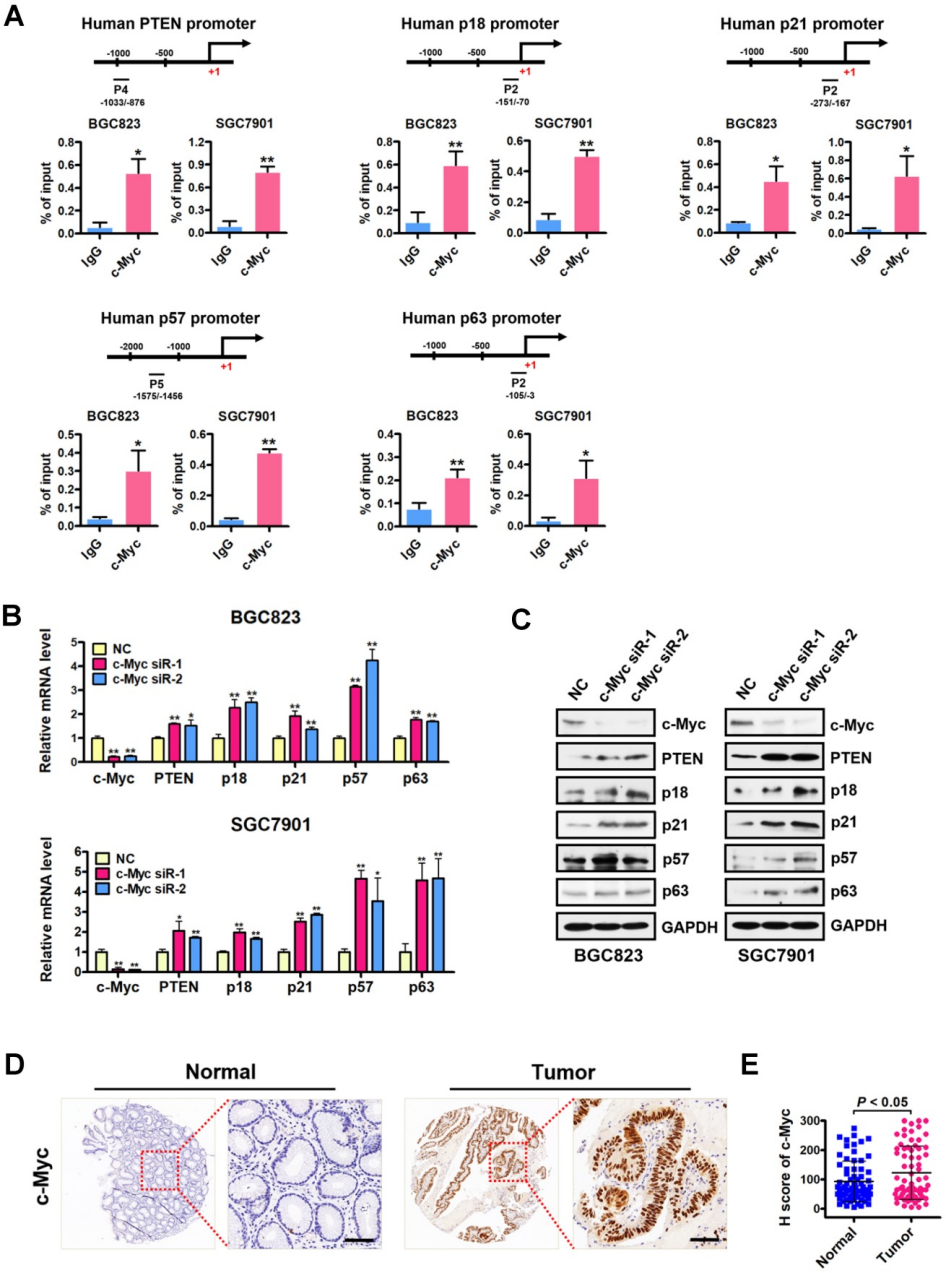
** c-Myc is co-enriched with H4R3me2s at PRMT5-targeted genes and represses their expression. (A)** c-Myc is enriched at the promoters of PTEN (P4), p18 (P2), p21 (P2), p57 (P5) and p63 (P2) in BGC823 and SGC7901 cells by ChIP analysis. IgG was used as a negative control. Data shown are mean ± SD (n = 3). *P < 0.05, **P < 0.01. **(B)** Relative mRNA expression levels of PTEN, p18, p21, p57 and p63 were analyzed by quantitative real-time PCR in negative control (NC) or c-Myc-silenced (c-Myc siR-1/2) BGC823 and SGC7901 cells. GAPDH was used as an endogenous control. Data shown are mean ± SD (n = 3). *P < 0.05, **P < 0.01. **(C)** Immunoblots of PTEN, p18, p21, p57 and p63 protein levels in negative control (NC) or c-Myc-silenced (c-Myc siR-1/2) BGC823 and SGC7901 cells. GAPDH served as a loading control. **(D)** Representative images of IHC staining of c-Myc in adjacent noncancerous (Normal) or gastric cancer (Tumor) tissues. The boxed areas in the left images are magnified in the right images. Scale bar: 50 µm. **(E)** IHC score of c-Myc in gastric cancer (n = 70) and adjacent noncancerous tissues (n = 70),* P* < 0.05.

We next examined the occupancy of c-Myc at the H4R3me2s-enriched region of PRMT5-targeted gene promoters in BGC823 cells under the same conditions as in Figure 3A. Consistently, ChIP assays showed a significant c-Myc enrichment at the promoters of PTEN, p18, p21, p57 and p63 (Figure 4A), further implicating c-Myc in the transcriptional repression of these PRMT5-targeted genes. To confirm the effect of c-Myc on those PRMT5-targeted genes, we silenced c-Myc expression in BGC823 and SGC7901 cell lines by transfecting with c-Myc-targeting siRNAs. We showed that PTEN, p18, p21, p57 and p63 mRNA levels and protein levels were all significantly upregulated when c-Myc was knocked down (Figure 4B-C). We further evaluated the protein levels of c-Myc in gastric cancer tissues using tissue arrays containing 90 pairs of gastric cancer and their matched non-tumorous tissues. After scoring c-Myc staining, we confirmed a significant upregulation of c-Myc in gastric cancer tissues, compared with that in normal tissues (Figure 4D-E). Taken together, these results indicate that c-Myc is co-enriched with H4R3me2s at the PRMT5-targeted genes and represses their expression.

### PRMT5-dependent direct interaction with c-Myc represses gene expression of PTEN and p57

**Figure 5:**
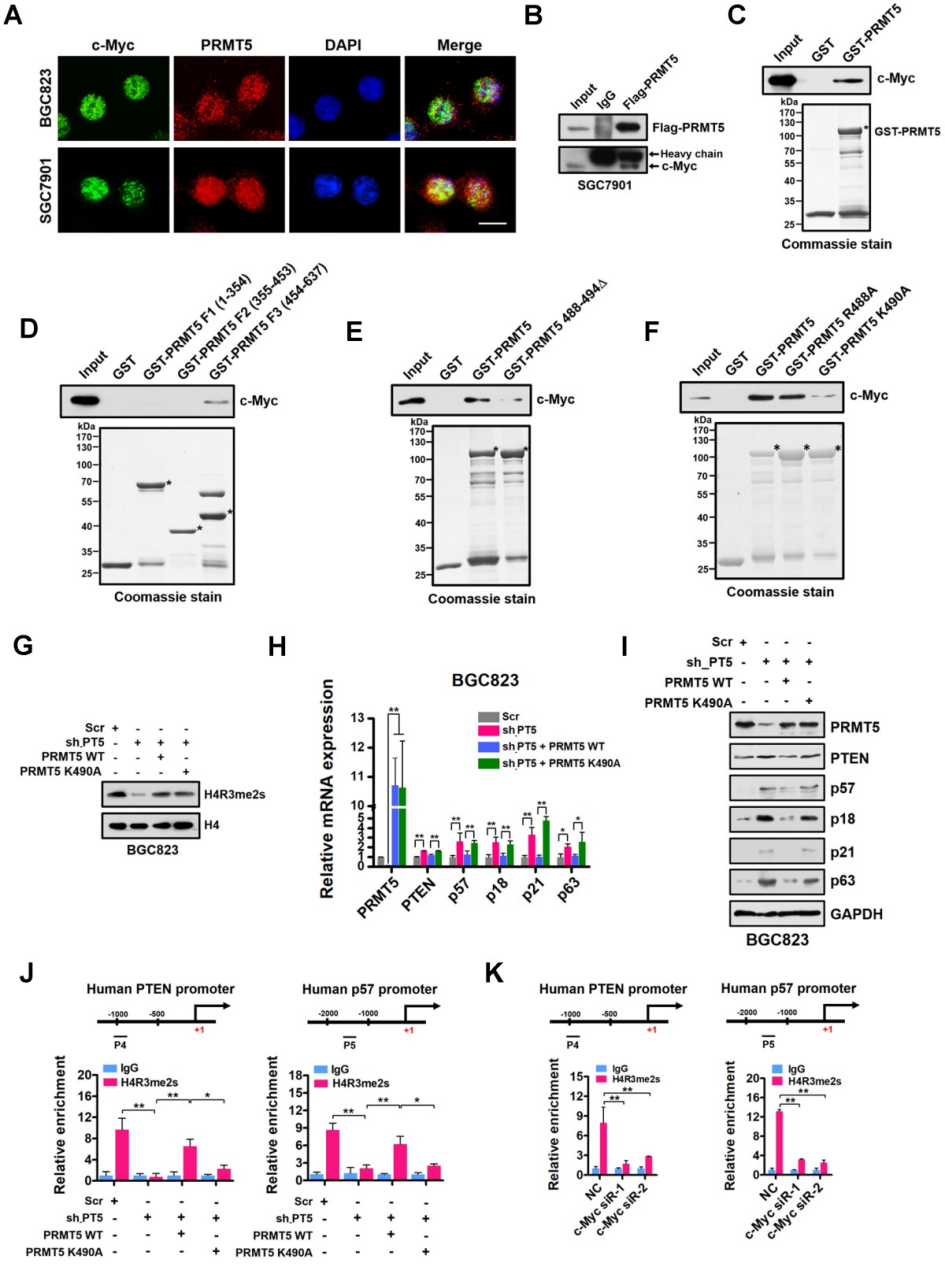
** PRMT5-dependent direct interaction with c-Myc represses gene expression of PTEN and p57. (A)** The subcellular location of c-Myc and PRMT5 proteins was documented in BGC823 and SGC7901 cells by immunofluorescence microscopy. Scale bar: 10 μm. **(B)** Co-immunoprecipitation of endogenous c-Myc with Flag-PRMT5 from SGC7901 cells overexpressing Flag-tagged PRMT5. IgG was used as the negative control. **(C)** Western blot analysis of c-Myc binding to purified GST or GST-PRMT5 fusion protein using c-Myc antibody (top). GST or GST-PRMT5 fusion protein purified from *E. coli* was visualized by Coomassie blue staining (bottom). An asterisk denotes the GST-PRMT5 fusion protein.** (D)** Western blot analysis of c-Myc binding to purified GST, GST-PRMT5 fragments F1 (amino acids 1-354), F2 (amino acids 355-453) or F3 (amino acids 454-637) using c-Myc antibody (top). GST or GST-PRMT5 F1, F2, or F3 fusion proteins from *E. coli* was visualized by Coomassie blue staining (bottom). Asterisks denote the GST-PRMT5 F1, F2, and F3 fusion proteins. **(E)** Western blot analysis of c-Myc binding to purified GST, GST-PRMT5 or GST-PRMT5 488-494Δ (deletion of amino acids 488-494) using c-Myc antibody (top). GST, GST-PRMT5 or GST-PRMT5 488-494Δ purified from *E. coli* was visualized by Coomassie blue staining (bottom). Asterisks denote the GST-PRMT5 or GST-PRMT5 488-494Δ fusion proteins. **(F)** Western blot analysis of c-Myc binding to purified GST, GST-PRMT5, GST-PRMT5 R488A or GST-PRMT5 K490A using c-Myc antibody (top). GST, GST-PRMT5, GST-PRMT5 R488A or GST-PRMT5 K490A purified from *E. coli* was visualized by Coomassie blue staining (bottom). Asterisks denote the GST- PRMT, GST-PRMT5 R488A or GST-PRMT5 K490A fusion proteins. **(G)** Western blot analysis of H4R3me2s levels in Scr, sh-PT5-treated, sh-PT5 + PRMT5 WT-treated or sh-PT5 + PRMT5 K490A-treated BGC823 cells. Histone H4 served as a loading control. **(H)** Relative mRNA levels of PRMT5, PTEN, p57, p18, p21 and p63 was examined by quantitative real-time PCR assays in Scr, sh-PT5-treated, sh-PT5 + PRMT5 WT-treated or sh-PT5 + PRMT5 K490A-treated BGC823 cells. GAPDH was used as an endogenous control. *P < 0.05, **P < 0.01. **(I)** Protein levels of PRMT5, PTEN, p57, p18, p21 and p63 were examined by Western blot assays in Scr, sh-PT5-treated, sh-PT5 + PRMT5 WT-treated or sh-PT5 + PRMT5 K490A-treated BGC823 cells. GAPDH served as a loading control. **(J)** Relative enrichment of H4R3me2s at the promoters of PTEN (P4, left panel) and p57 (P5, right panel) was examined by ChIP assays in Scr, sh-PT5-treated, sh-PT5 + PRMT5 WT-treated or sh-PT5 + PRMT5 K490A-treated BGC823 cells. IgG was used as a negative control. Data shown are mean ± SD (n = 3). *P < 0.05, **P < 0.01. **(K)** Relative enrichment of H4R3me2s at the promoters of PTEN (P4, left panel) and p57 (P5, right panel) was examined by ChIP assays in NC, c-Myc siR-1 and c-Myc siR-2-treated BGC823 cells. IgG was used as a negative control. Data shown are mean ± SD (n = 3). **P < 0.01.

Given that PRMT5-mediated H4R3me2s is enriched at the E-box motif and that c-Myc co-enriched with H4R3me2s at the PRMT5-targeted genes regulates their expression, we considered that PRMT5 and c-Myc directly interact. To test this, we first performed immunofluorescence microscopy and observed co-localization of PRMT5 and c-Myc in gastric cancer BGC823 and SGC7901 cells. We found that c-Myc co-localized with PRMT5 dominantly in the nucleus of BGC823 and SGC7901 cells (Figure 5A). Next, we carried out co-immunoprecipitation (co-IP) experiments using anti-Flag antibody-conjugated sepharose beads to precipitate Flag-tagged PRMT5. Associated proteins from cellular extracts of SGC7901 cells overexpressing Flag-tagged PRMT5 was detected by Western blot. As shown in Figure 5B, endogenous c-Myc was co-precipitated with Flag-PRMT5, implying an association between PRMT5 and c-Myc in gastric cancer cells. Thirdly, a GST pull-down assay demonstrated that purified His-tagged c-Myc could be pulled down by GST-PRMT5 (as bait protein) whereas GST alone showed no interaction, suggesting a direct interaction of PRMT5 and c-Myc proteins* in vitro* (Figure 5C).

Next, we mapped the region in PRMT5 responsible for binding to c-Myc. The full-length human PRMT5 protein was divided into three fragments, amino acids 1-354 (F1), 355-453 (F2) and 454-637 (F3), according to its subdomain structure. Purified His-tagged c-Myc protein pre-adsorbed to nitrilotriacetic acid-nickel beads was incubated with purified GST or GST fusion PRMT5 proteins containing fragments 1-354 (F1), 355-453 (F2) or 454-637 (F3) respectively. We found that only fragment 3 (F3) of PRMT5 was able to interact with c-Myc (Figure 5D).

In order to probe how c-Myc interacts with PRMT5, we constructed a deletion mutant in which amino acids 488-494 in PRMT5 were removed (PRMT5 488-494Δ). Interestingly, GST pull-down assays showed that the direct interaction between this PRMT5 deletion mutant and c-Myc was largely lost (Figure 5E). A single point mutation in WT PRMT5 K490A (K to A at aa490) resulted in a significant decrease in the interaction whereas PRMT5 R488A (K to A at aa488) retained the ability to interact with c-Myc (Figure 5F), indicating that K490 of PRMT5 is essential for the interaction of PRMT5 with c-Myc.

To investigate the impact of the K490A mutation on expression of PRMT5-targeted genes, we overexpressed wildtype PRMT5 or the PRMT5 K490A mutant in PRMT5-depleted BGC823 cells. Of note, the PRMT5 K490A mutant displayed the same methyltransferase activity as the wildtype PRMT5 (Figure 5G). However, overexpression of the PRMT5 K490A mutant in PRMT5-depleted BGC823 cells failed to repress PTEN, p57, p18, p21 and p63 expression in contrast to that of wildtype PRMT5 (Figure 5H-I). Interestingly, we found that the occupancies of H4R3me2s on the PTEN and p57 promoters in BGC823 cells overexpressing the PRMT5 K490A mutant were also significantly lower than in cells overexpressing wildtype PRMT5 (Figure 5J). However, the occupancies of c-Myc on the PTEN and p57 promoters were not significantly changed when PRMT5 K490A mutant was overexpressed in PRMT5-depleted BGC823 cells (Figure S3), suggesting that c-Myc binding to these promoters was independent of the interaction with PRMT5. Notably, further ChIP assays showed a significant reduction of H4R3me2s enrichment at the promoters of PTEN and p57 genes when c-Myc expression was silenced by siRNAs in BGC823 cells (Figure 5K). Together, these results suggest that PRMT5 is recruited to promoters via c-Myc interaction, where it mediates H4R3me2s modifications and transcriptional repression.

### Downregulated PTEN and p57 expression levels in primary human gastric cancer tissues correlate inversely with expression levels of PRMT5 and associate with poor clinical outcomes

**Figure 6:**
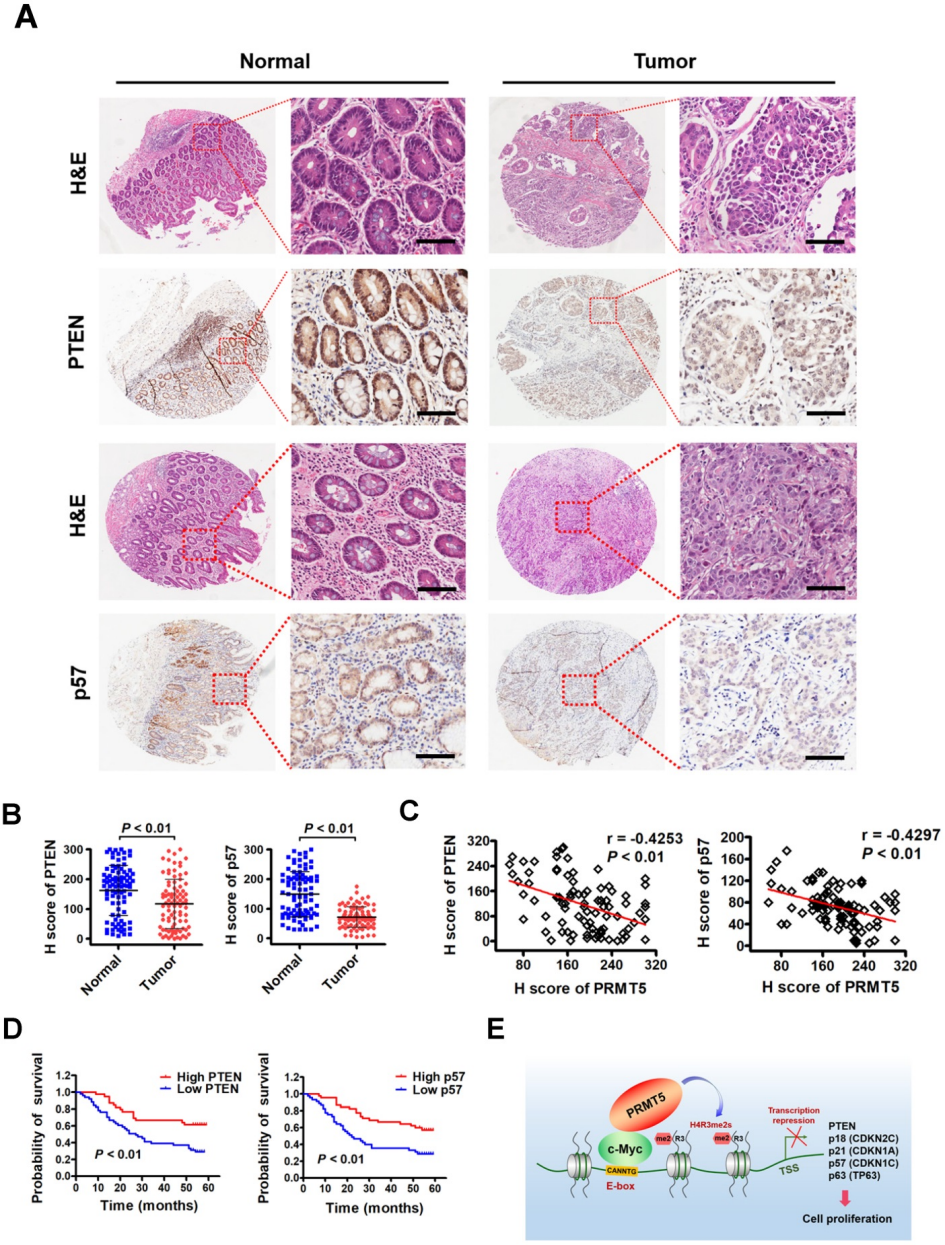
** Downregulated PTEN and p57 expression levels in primary human gastric cancer tissues correlate inversely with expression levels of PRMT5 and associate with poor clinical outcomes. (A)** Representative images of H&E and IHC staining of PTEN and p57 in adjacent noncancerous (n = 90) and gastric cancer (n = 90) tissues. The boxed areas in the left images are magnified in the right images. Scale bar: 50 µm. **(B)** IHC scores of PTEN (left panel) and p57 (right panel) in adjacent noncancerous (n = 90) and gastric cancer (n = 90) tissues. *P* < 0.01. **(C)** Left panel, correlations of PTEN and PRMT5 protein levels in gastric cancer tissues (n = 90) was performed by Spearman's correlation analysis (r = -0.4253, *P* < 0.01). Right panel, correlations of p57 and PRMT5 protein levels in gastric cancer tissues (n = 90) was performed by Spearman's correlation analysis (r = -0.4297, *P* < 0.01). **(D)** Left panel, overall survival of High PTEN (n = 45) and Low PTEN (n = 45) gastric cancer patients was performed by Kaplan-Meier survival analysis, *P* < 0.01. Right panel, overall survival of High p57 (n = 45) and Low p57 (n = 45) gastric cancer patients was performed by Kaplan-Meier survival analysis, *P* < 0.01. **(E)** Model for PRMT5-dependent role in regulation of c-Myc target gene expression. PRMT5- mediated H4R3me2s epigenetically represses transcription of c-Myc target genes, PTEN, p18, p21, p57 and p63, to promote cell proliferation and gastric cancer progression.

To investigate the clinical significance of PTEN and p57 expression in patients with gastric cancer, we first examined their expression by immunohistochemical staining (IHC) on a tissue array containing 90 pairs of gastric cancer samples and their matched non-tumorous tissues. We found that PTEN and p57 protein were significantly downregulated in tumor tissues compared with matched adjacent normal tissues (Figure 6A-B). Notably, levels of PTEN (r = -0.4253, *P* < 0.01) or p57 (r = -0.4297, *P* < 0.01) exhibited a significant inverse correlation with PRMT5 levels in these gastric cancer samples calculated by Pearson correlation (Figure 6C). Moreover, Kaplan-Meier survival analysis showed that low PTEN and p57 expression was correlated with poor overall survival (Figure 6D). These results indicate that downregulated PTEN and p57 expression levels correlate with expression levels of PRMT5 in human gastric cancer tissues, and associate with poor prognosis in gastric cancer, further supporting an important regulatory role for PRMT5 during gastric cancer progression.

## Discussion

In this study, we found that c-Myc could interact directly with PRMT5 to transcriptionally repress the expression of a cohort of genes, including PTEN, CDKN2C (p18^INK4C^), CDKN1A (p21^CIP1/WAF1^), CDKN1C (p57^KIP2^) and p63, to promote gastric cancer cell growth (Figure 6E). Although there have been some preliminary reports of an association between c-Myc and PRMT5 in neuroblastoma cells and glioblastoma cells 47, 48, this is the first report of the mechanism and significance of c-Myc interaction with PRMT5 in gastric cancer cells. Several mechanisms of Myc-mediated repression have been proposed previously. The first is dependent on a transcriptional initiator (Inr) element and involves a zinc-finger transcription factor, Miz-1, which binds to the Inr element. Myc forms a complex with Miz-1 and thereby becomes activated to repress gene expression 46, 49. The other mechanism is transcription factor Sp1-dependent: c-Myc interacts with the Sp1 or Sp1/Smad complex, thus inhibiting the recruitment of other positive regulators to promoters of target genes 50. In recent years, accumulating evidence has also implied that noncoding RNAs may play critical roles in c-Myc-mediated transcription repression 8. And another mechanism of repression involves polycomb repressive complex (PRC) associated with methylation of lysine 27 at histone H3 (H3K27me3), i.e., the PTEN-AKT-EZH2 pathway, to regulate repressed target genes indirectly 51. In the current study, we find an alternative mechanism: c-Myc co-enriches with PRMT5-mediated H4R3me2s at promoters of target genes to repress their expression. This is reminiscent of Myc's association to Sin3/HDAC complexes mediated by the MXD/MNT family members to compete for MAX binding at E-box motif to repress its target gene transcription 52. However, the action of gene repression by c-Myc/PRMT5 in this context is direct. Of note, the extent of transcriptional regulation of those c-Myc- targeted genes varies, and the altered levels of transcription do not match the altered levels of their proteins. This suggests that c-Myc may bind to other transcriptional machineries or epigenetic regulators in addition to PRMT5 to modulate these gene activities. Interestingly, two prototypical Myc-repressed genes, CDKN1B (p27^KIP1^) and CDKN2B (p15^INK4B^) 39, 46, 53, 54, showed no response to manipulation of c-Myc in the context of gastric cancer (Figure 2A), strengthening the concept that c-Myc regulates gene expression selectively and specifically.

Through the analysis of publicly available H4R3me2s ChIP-Seq datasets, we found that a DNA motif, CAGCTG, was indeed observed in the H4R3me2s-enriched regions and exactly corresponds to an E-box variant element that c-Myc binds, suggesting it may also play a functional role in PRMT5-mediated transcriptional repression. Subsequent ChIP assays showed that c-Myc was co-enriched with H4R3me2s at the PRMT5-targeted genes and repressed their expression, suggesting that PRMT5-mediated H4R3me2s may have a preferential neighboring E-box or its variants. Of note, previously Guo *et al*. provided evidence proposing a perspective for c-Myc function where the transcription machinery rather than DNA sequence elements plays a major role in recruiting the c-Myc-Max heterodimer to genomic sites 55. The sites occupied by c-Myc-MAX heterodimer across the human genome correlate with the RNA Pol II transcription machinery rather than with E-box elements* in vitro* 55. Although a majority of studies reported that the genome occupancy of c-Myc-MAX heterodimer is thought to be driven by E-box elements near TSS 11, 56, 57, we cannot exclude the possibility that the depositions of H4R3me2s and c-Myc at promoters of PRMT5- targeted genes might be a consequence of the RNA Pol II transcription machinery and E-boxes.

As an epigenetic regulator, PRMT5 could transcriptionally regulate the expression of a wide spectrum of cellular events, including cell growth/ proliferation 58-60, cell invasion/metastasis 61, altered DNA replication and genomic instability 62 and misregulation of cell cycle progression 29, 63. Our results are consistent with the observation by Kanda and coworkers in which PRMT5 was positively correlated with poor survival in gastric cancer 22. In a recent report, Liu *et al.* found that PRMT5-mediated IRX1 inactivation plays an important role in promoting tumorigenicity and metastasis of gastric cancer cells 23. We note that the mechanism of recruitment of PRMT5 to the promoter of the target gene was not disclosed and the conclusion was based on monitoring the expression of a single target gene. Similarly, in agreement with our results, recent studies found that PRMT5-mediated H4R3me2s in the promoter regions of target genes led to transcription repression of the cyclin-dependent cell cycle inhibitor CDKN1A (p21^CIP1/WAF1^) as well as the tumor repressor PTEN in other malignancies 64, 65. Our current study revealed that cell cycle negative regulators p18, p21, p57, and tumor repressors PTEN and p63 are epigenetic targets of PRMT5 in gastric cancer. We found that PRMT5-mediated H4R3me2s is enriched at the E-box motif of these target genes, which binds the transcription factor c-Myc, delineating the molecular process of transcriptional repression.

Interestingly, previous reports suggested PRMT5 and N-Myc proteins physically interact and N-Myc protein stability is regulated by PRMT5 in neuroblastoma cells 47. Favia *et al.* showed that PRMT1 associates with Myc/PRMT5 in both HEK293T cells and glioblastoma stem cells and that Myc is both symmetrically and asymmetrically dimethylated by PRMT5 and PRMT1, respectively 66. Moreover, Tikhanovich *et al.* demonstrated that PRMT1 is necessary for c-Myc-dependent transcription through altering promoter recruitment of acetyltransferase p300 67. A recent report by Nagendra *et al.* suggested that PRMT5 physically associated with c-Myc and posttranslationally modulated the stability of c-Myc 68. In the current study, an interaction between PRMT5 and c-Myc is key to this gene regulation, as a single mutation in PRMT5 (K490A) rendered it unable to interact with c-Myc albeit retains its methyltransferase activity. Given the fact that PRMT5 inhibition may exhibit severely impaired cytokine signaling as well as elevation of p53 or other downstream targets, PRMT5 targeted therapy with PRMT5 inhibitors may potentially result in unpredictable outcomes, including severe side effects on essential B- or T-cell functions 69-71. Therefore, our work indicates that the c-Myc/PRMT5 interface may be a key potential point for developing small molecule inhibitors in c-Myc-driven tumors.

Cellular localization of PRMT5 helps dictate its role in cells. It is interesting that we observed a dominant nuclear localization of PRMT5 in both gastric cancer tissues and cells, although a small fraction was distributed in the cytoplasm as well. PRMT5 has been shown to predominantly localize in the cytoplasm in lung 72, prostate 73 and melanoma cancers 74. Mongiardi *et al.* observed a diffused cellular localization of PRMT5 both in the cytoplasm and nucleus of glioblastoma cells 48. Thus, PRMT5 localizes in both the nucleus and cytoplasm. Why PRMT5 is preferentially localized in the nucleus of gastric cancer cells remains to be explained. One possible reason is that PRMT5 may be capable of shuttling between the nucleus and cytoplasm, thus contributing to tumorigenesis and cancer progression 73, 75, 76. In fact, previously we have observed this phenomenon during erythroid cell differentiation 43. However, currently little is known about how PRMT5 is shuttled between the nucleus and cytoplasm under either pathological or physiological conditions.

Our study demonstrated that PRMT5-dependent epigenetic repression of c-Myc target genes regulates gastric cancer progression. However, we still do not understand how PRMT5-mediated H4R3me2s is fully recognized and thereby affects other epigenetic modifications to repress downstream gene expression and promote tumor progression. Large-scale cohort studies and detailed ChIP-seq analysis of PRMT5- mediated H4R3me2s and other histone marks would facilitate understanding their roles in transcriptional repression and gastric cancer progression. Nevertheless, our findings reveal important insights that link PRMT5-dependent transcription repression of c-Myc target genes and gastric cancer progression. Collectively, we unraveled a novel mechanism by which PRMT5-dependent transcriptional repression of c-Myc target genes is required for gastric cancer progression. Our results will help contribute to the development of new therapeutic strategies for gastric cancer in the future.

## Supplementary Material

Supplementary figures and tables.

## Abbreviations

PRMT5: protein arginine methyltransferase 5
H4R3me2s: symmetric di-methylation of histone H4 on Arg 3
SAM: S-adenosyl methionine
PTMs: Post-translational modifications
PRC: Polycomb repressive complex
bHLH: Basic helix-loop-helix
RT-PCR: Quantitative reverse transcription PCR
ChIP: Chromatin immunoprecipitation
HOMER: Hypergeometric Optimization of Motif EnRichment
Co-IP: Co-immunoprecipitation
IHC: Immunohistochemistry
H&E staining: Hematoxylin-eosin staining
