# Supplementary material for: PRMT5-dependent transcriptional repression of c-Myc target genes promotes gastric cancer progression
Source: Theranostics. 2020 Mar 15;10(10):4437–52. doi: 10.7150/thno.42047 (PMC7150477; doi:10.7150/thno.42047)
Supplement: Supplementary file 1 — Supplementary figures and tables. [file thnov10p4437s1.pdf]

Supplementary data

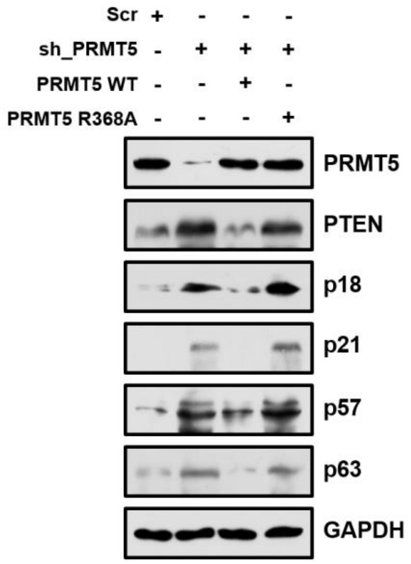

Figure S1. Immunoblots of PRMT5, PTEN, p18, p21, p57 and p63 levels in Scr, sh-PRMT5-treated, sh-PRMT5 + PRMT5 (wild type)-treated or sh-PRMT5 + PRMT5 R368A (enzymatically inactive)-treated BGC823 cells. GAPDH served as a loading control.

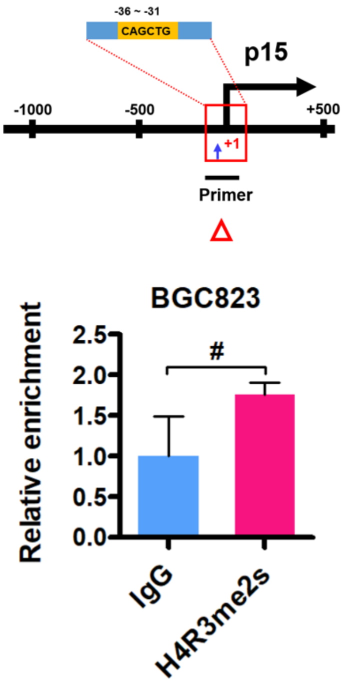

Figure S2. H4R3me2s enrichment at the proximal promoter region of p15 (-163 ~ +220) determined by ChIP analysis. IgG was used as a negative control. Data shown are mean ± SD (n = 3). $^{\#}P > 0.05$. The CAGCTG motif is shown in the promoter region of p15 (up panel).

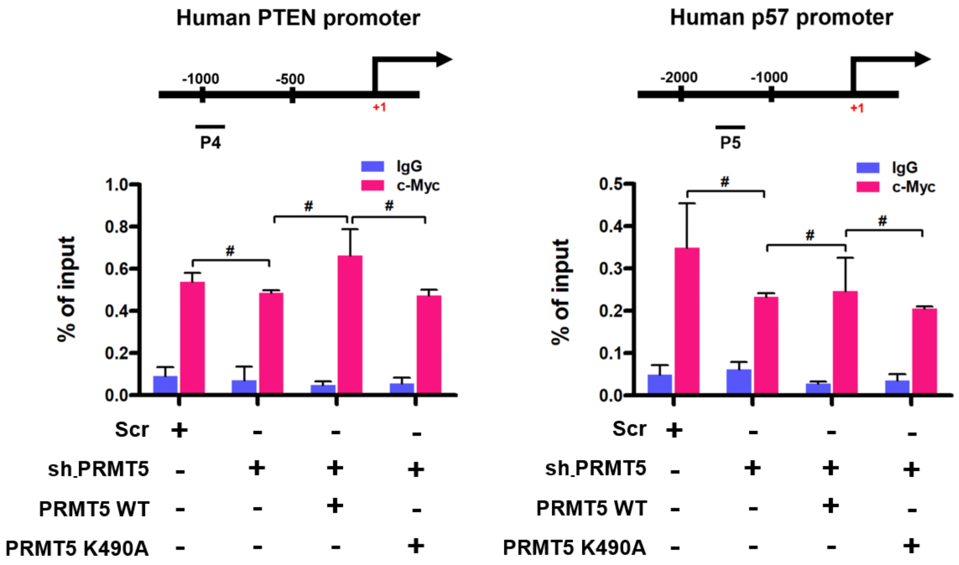

Figure S3. Relative enrichment of c-Myc at the promoters of PTEN (P4, left panel) and p57 (P5, right panel) was examined by ChIP assays in Scr, sh-PRMT5-treated, sh-PRMT5 + PRMT5 WT-treated or sh-PRMT5 + PRMT5 K490A-treated BGC823 cells. IgG was used as a negative control. Data shown are mean ± SD (n = 3). $^{\#}P > 0.05$.

Table S1. Real-time PCR primer sequences

CDKN1A forward (5'-3')	TACCCTTGTGCCTCGCTCAG
CDKN1A reverse (5'-3')	CGGCGTTTGGAGTGGTAGA
PRMT5 forward (5'-3')	TCAGGAAGATAACACCAACCTGG
PRMT5 reverse (5'-3')	AGCCACTGCAATCCTCTTACTAT
GAPDH forward (5'-3')	GAGCCACATCGCTCAGACAC
GAPDH reverse (5'-3')	CATGTAGTTGAGGTCAATGAAGG
TP53 forward (5'-3')	GAGGTTGGCTCTGACTGTACC
TP53 reverse (5'-3')	TCCGTCCCAGTAGATTACCAC
ABL1 forward (5'-3')	CCAGGTGTATGAGCTGCTAGAG
ABL1 reverse (5'-3')	GTCAGAGGGATTCCACTGCCAA
ANAPC2 forward (5'-3')	CAGGACAGTGAGGATGACTCAG
ANAPC2 reverse (5'-3')	TTGCTGCCGTAGATGCTGACCA
ANAPC4 forward (5'-3')	AAGGAGGTGACGTGTCTGGCAT
ANAPC4 reverse (5'-3')	GCATACAGGAAACTGGAGCCTC
DIRAS3 forward (5'-3')	CACATCACCGACAGCAAGAGTG
DIRAS3 reverse (5'-3')	CAGGGTTTCCTTCTTGGTGACTG
ATM forward (5'-3')	TGTTCCAGGACACGAAGGGAGA
ATM reverse (5'-3')	CAGGGTTCTCAGCACTATGGGA
ATR forward (5'-3')	GGAGATTTCCTGAGCATGTTCGG
ATR reverse (5'-3')	GGCTTCTTTACTCCAGACCAATC
BCCIP forward (5'-3')	GAAGAGGACGAGGTCATTGACG
BCCIP reverse (5'-3')	GCAGTGTTCACAGGAGCCTTTAG
BCL2 forward (5'-3')	ATCGCCCTGTGGATGACTGAGT
BCL2 reverse (5'-3')	GCCAGGAGAAATCAAACAGAGGC

BRCA2 forward (5’-3’)	GGCTTCAAAAAGCACTCCAGATG
BRCA2 reverse (5’-3’)	GGATTCTGTATCTCTTGACGTTCC
CCNB1 forward (5’-3’)	GACCTGTGTCAGGCTTTCTCTG
CCNB1 reverse (5’-3’)	GGTATTTTGGTCTGACTGCTTGC
CCNB2 forward (5’-3’)	CAACCAGAGCAGCACAAGTAGC
CCNB2 reverse (5’-3’)	GGAGCCAACTTTTCCATCTGTAC
CCNC forward (5’-3’)	GCAGAAAGATGCCAGGCAATGG
CCNC reverse (5’-3’)	CTCTCATCGAAATTCTTCCACTGC
CCND forward (5’-3’)	TCTACACCGACAACTCCATCCG
CCND reverse (5’-3’)	TCTGGCATTTTGGAGAGGAAGTG
CCND2 forward (5’-3’)	GAGAAGCTGTCTCTGATCCGCA
CCND2 reverse (5’-3’)	CTTCCAGTTGCGATCATCGACG
CCNE1 forward (5’-3’)	TGTGTCCTGGATGTTGACTGCC
CCNE1 reverse (5’-3’)	CTCTATGTCGCACCACTGATACC
CCNF forward (5’-3’)	CTGCGTCTTGAGCCTCCATAAG
CCNF reverse (5’-3’)	CCTGGCTGATTTCTCCATAGCG
CCNH forward (5’-3’)	CGATGTCATTCTGCTGAGCTTGC
CCNH reverse (5’-3’)	TCTACCAGGTCGTCATCAGTCC
CCNT1 forward (5’-3’)	TGTTGACGCCACTGTGACCTTG
CCNT1 reverse (5’-3’)	GTTTTCTTGGCAGCCTCGCATG
CCNT2 forward (5’-3’)	GGCTGCAAAAGTGGAAGAACAGG
CCNT2 reverse (5’-3’)	CCAGTTCTTGAGTCTGTTGAAGG
CDC16 forward (5’-3’)	GTGTCTTGGTTTGCAGTGGGATG
CDC16 reverse (5’-3’)	GTGCTCACTCTCCACCGCAAAT

CDK1 forward (5'-3')	GGAAACCAGGAAGCCTAGCATC
CDK1 reverse (5'-3')	GGATGATTCAGTGCCATTTTGCC
CDC20 forward (5'-3')	CGGAAGACCTGCCGTTACATTC
CDC20 reverse (5'-3')	CAGAGCTTGCACTCCACAGGTA
CDK2 forward (5'-3')	ATGGATGCCTCTGCTCTCACTG
CDK2 reverse (5'-3')	CCCGATGAGAATGGCAGAAAGC
CDK4 forward (5'-3')	CCATCAGCACAGTTCGTGAGGT
CDK4 reverse (5'-3')	TCAGTTCGGGATGTGGCACAGA
CDK5R1 forward (5'-3')	TCATCTCCGTGCTGCCTTGGAA
CDK5R1 reverse (5'-3')	CTCATTGTTGAGGTGCGTGATGT
CDK6 forward (5'-3')	GGATAAAGTTCCAGAGCCTGGAG
CDK6 reverse (5'-3')	GCGATGCACTACTCGGTGTGAA
CDK7 forward (5'-3')	GCACACCAACTGAGGAACAGTG
CDK7 reverse (5'-3')	AAGTCGTCTCCTGCTGCACTGA
CDK8 forward (5'-3')	GCTGATAGGAAGGTGTGGCTTC
CDK8 reverse (5'-3')	CCGAGGTAACTGAACTGGCTTC
CDKN1B forward (5'-3')	ATAAGGAAGCGACCTGCAACCG
CDKN1B reverse (5'-3')	TTCTTGGGCGTCTGCTCCACAG
CKS1B forward (5'-3')	GGAATCTTGGCGTTCAGCAGAG
CKS1B reverse (5'-3')	GAGGCTGAAAAGTAGCTTGCCAG
DDX11 forward (5'-3')	CCTTTGGCAAGGATGTTCGGCT
DDX11 reverse (5'-3')	TGTCCACACAGCGGTCGTTGAT
E2F4 forward (5'-3')	GGAAGGTATCGGGCTAATCGAG
E2F4 reverse (5'-3')	AGCTCCTCGATCTCTGCCTTGA

GADD45A forward (5'-3')	CTGGAGGAAGTGCTCAGCAAAG
GADD45A reverse (5'-3')	AGAGCCACATCTCTGTCGTCGT
KNTC1 forward (5'-3')	GTCTTTGCCTCCTGCTGAAGCT
KNTC1 reverse (5'-3')	TCCACGGATGTCTTCGCTACAG
MKI67 forward (5'-3')	GAAAGAGTGGCAACCTGCCTTC
MKI67 reverse (5'-3')	GCACCAAGTTTTACTACATCTGCC
RAD9A forward (5'-3')	TCACTGGCGATGCTGGAGAAGA
RAD9A reverse (5'-3')	GACTCACAGTCCTGGAAGGACA
RB1 forward (5'-3')	CAGAAGGTCTGCCAACACCAAC
RB1 reverse (5'-3')	TTGAGCACACGGTCGCTGTTAC
SKP2 forward (5'-3')	GATGTGACTGGTCGGTTGCTGT
SKP2 reverse (5'-3')	GAGTTCGATAGGTCCATGTGCTG
TFDP1 forward (5'-3')	CACTTTGCCTCTCAGAACCAGC
TFDP1 reverse (5'-3')	CTTTCCTCTGCACCTTCTCGCA
TFDP2 forward (5'-3')	CCTGGTGCCAAAGGCTTTAGAAG
TFDP2 reverse (5'-3')	TGGCACCAGTGGTCAGGTCTAA
CDKN2C forward (5'-3')	CGTCAATGCACAAAATGGATTTGG
CDKN2C reverse (5'-3')	GAATGACAGCGAAACCAGTTCGG
CDKN2A forward (5'-3')	CTCGTGCTGATGCTACTGAGGA
CDKN2A reverse (5'-3')	GGTCGGCGCAGTTGGGCTCC
CDKN2B forward (5'-3')	ACGGAGTCAACCGTTTCGGGAG
CDKN2B reverse (5'-3')	GGTCGGGTGAGAGTGGCAGG
CDKN3 forward (5'-3')	ATGGAGGGACTCCTGACATAGC
CDKN3 reverse (5'-3')	TCTCCCAAGTCCTCCATAGCAG

CHEK1 forward (5'-3')	GTGTCAGAGTCTCCCAGTGGAT
CHEK1 reverse (5'-3')	GTTCTGGCTGAGAACTGGAGTAC
CHEK2 forward (5'-3')	GACCAAGAACCTGAGGAGCCTA
CHEK2 reverse (5'-3')	GGATCAGATGACAGCAGGAGTTC
TP63 forward (5'-3')	CAGGAAGACAGAGTGTGCTGGT
TP63 reverse (5'-3')	AATTGGACGGCGGTTCATCCCT
PTEN forward (5'-3')	TGAGTTCCCTCAGCCGTTACCT
PTEN reverse (5'-3')	GAGGTTTCCTCTGGTCCTGGTA
c-Myc forward (5'-3')	CCTGGTGCTCCATGAGGAGAC
c-Myc reverse (5'-3')	CAGACTCTGACCTTTTGCCAGG
p57 forward (5'-3')	GCGTCCCTCCGCAGCACAT
p57 reverse (5'-3')	GGTTCTGGTCCTCGGCGTTCA

Table S2. Real-time PCR primer sequences for ChIP

PTEN P1 forward (5’-3’)	GTGGCGGGACTCTTTATG
PTEN P1 reverse (5’-3’)	CGGCTCAACTCTCAAACT
PTEN P2 forward (5’-3’)	GCATTTCCCTCTACACTGA
PTEN P2 reverse (5’-3’)	GTGGAGGACTGATGATGAA
PTEN P3 forward (5’-3’)	TTCAACGGCTATGTGTTCA
PTEN P3 reverse (5’-3’)	TGTCCTCATGGTGTCAGT
PTEN P4 forward (5’-3’)	GTGCTTGTGTAACCAGTTC
PTEN P4 reverse (5’-3’)	GAGGCAAGTCGTCTTCTT
PTEN P5 forward (5’-3’)	CGATCCAACTCTCAGCATT
PTEN P5 reverse (5’-3’)	CTCAGCCAAGTGACTTATCT
PTEN P6 forward (5’-3’)	CGCTAGGTCTCTTGAGGT
PTEN P6 reverse (5’-3’)	AGGTTGAAGCACTGAGTTG
p18 P1 forward (5’-3’)	GGGAAAGGAAGGAAAGGACAGCG
p18 P1 reverse (5’-3’)	TGATGCGGAAAGCGTCTACGG
p18 P2 forward (5’-3’)	AGACTTGACGGGAGGAGGTGGAG
p18 P2 reverse (5’-3’)	GCTCGCAGTCTCGCACGCTC
p18 P3 forward (5’-3’)	CTGAGGAACGACTCCCTTTATGCC
p18 P3 reverse (5’-3’)	GGCGAGGACAGGGGTTTGTTATTT
p18 P4 forward (5’-3’)	GAGAACTTCGGCAACCAA
p18 P4 reverse (5’-3’)	GACTGGAAACTGCGAAATAG
p18 P5 forward (5’-3’)	TCCCTCTACCTACCAATC
p18 P5 reverse (5’-3’)	AGCAGTATCTGTACCCTTC
p18 P6 forward (5’-3’)	CCTTCTGAACTAAGACCCTA

p18 P6 reverse (5'-3')	TTTCCCATGATGGCTACT
p21 P1 forward (5'-3')	GTTTCTGCGGCAGGTGAAT
p21 P1 reverse (5'-3')	GGGAGCGTGACCAGGGAT
p21 P2 forward (5'-3')	TGTGTCCTCCTGGAGAGTGC
p21 P2 reverse (5'-3')	CAGTCCCTCGCCTGCGTTG
p21 P3 forward (5'-3')	TTCCCGGAAGCATGTGACAAT
p21 P3 reverse (5'-3')	CACTAGGTCACCTCTCCCAGA
p21 P4 forward (5'-3')	AGGTAGATGGGAGCGGATAGA
p21 P4 reverse (5'-3')	ACCCTCATTTGCAGATGGTTT
p21 P5 forward (5'-3')	CATTTGACAACCAGCCCTTT
p21 P5 reverse (5'-3')	TGGGAGGACACAGTAGCAGA
p21 P6 forward (5'-3')	CAGCAGATCCTTGCGACAG
p21 P6 reverse (5'-3')	CCATGCACTTGAATGTGTACC
p57 P1 forward (5'-3')	GTCCCTCCGCAGCACATCCACG
p57 P1 reverse (5'-3')	CGGTTCTGGTCCTCGGCGTTCA
p57 P2 forward (5'-3')	CAGGCCAAGTGCGCTGTGCTCG
p57 P2 reverse (5'-3')	GGTGGACTCTTCTGCGTCGGGTTCG
p57 P3 forward (5'-3')	GCTGGCAGCGGCGGGTCCAA
p57 P3 reverse (5'-3')	TCCGGCCAGGCCCAACTCGA
p57 P4 forward (5'-3')	ACCGAGGTCTGTACCAGGAAGGG
p57 P4 reverse (5'-3')	CAGGCTGGCTGGAAGCTGTTGT
p57 P5 forward (5'-3')	TGGCTTTTGGTTCCACCATC

p57 P5 reverse (5’-3’)	TCTTCTGGGTCTCAGGGAAG
p57 P6 forward (5’-3’)	GTTCTGAGCCCTGCTTCCTT
p57 P6 reverse (5’-3’)	TGTAGCTGCCATCATCCTGTG
p63 P1 forward (5’-3’)	CTGCCCTGACCCTTACAT
p63 P1 reverse (5’-3’)	GCTGACTAAACAAGGAGGA
p63 P2 forward (5’-3’)	TTGGCTAAAATCAAGAAAC
p63 P2 reverse (5’-3’)	GCTAAAAGCAATAGGGTCA
p63 P3 forward (5’-3’)	AGCAGGGTGGACACTCATC
p63 P3 reverse (5’-3’)	TGTTGTTGGCAATTTGGA
p63 P4 forward (5’-3’)	AAGACATAAAGAATAGAGTGGAGCCG
p63 P4 reverse (5’-3’)	TTTGCCTGACCCGAATAA
p63 P5 forward (5’-3’)	AGAAATCTTCACTCCACCTT
p63 P5 reverse (5’-3’)	AATCAAACAGCTTGCTCC
p63 P6 forward (5’-3’)	GTGGCTCATGCCTGTAATC
p63 P6 reverse (5’-3’)	GTTGCCCTGACTGGTCTC
p15 Primer forward (5’-3’)	TGCGTCCTAGCATCTTTGG
p15 Primer reverse (5’-3’)	ACCCTCCCGTCGTCCTT

Table S3. Clinicophathologic characteristics of PRMT5 expression in gastric cancer patients

Characteristics	Cases	H score of PRMT5 (Mean±SD)	*P* value[a]
	90		
Gender			
**Male**	62	190.89±65.38	*0.4673*
**Female**	28	180.61±53.00	
Age			
**>60**	52	186.48±65.23	*0.8293*
**≤60**	38	189.34±57.28	
Tumor size			
**≥5 cm**	53	188.43±55.30	*0.8918*
**<5 cm**	37	186.62±70.61	
Tumor stage[b]			
**I-II**	15	154.13±70.71	*0.0201*
**III-IV**	75	194.40±57.93	
Lymph node status[b]			
**N0**	21	174.76±73.95	*0.2751*
**N1-3**	69	191.62±57.48	
Distant metastasis[b]			
**M0**	88	187.47±62.17	*0.8215*
**M1**	2	197.50±45.96	

[a]*P* values were derived using Student's *t* test to compare values for the two parameters in each category.
[b]The tumor stage, lymph node status, and metastasis were classified according to the international system for staging gastric cancer[1].

Reference:

1. In H, Solsky I, Palis B, Langdon-Embry M, Ajani J, Sano T. Validation of the 8th Edition of the AJCC TNM Staging System for Gastric Cancer using the National Cancer Database.Ann Surg Oncol. 2017;24(12):3683-3691.

Table S4. Motifs distributed in the H4R3me2s-enriched binding regions ($P < 10^{-12}$) obtained from the ChIP-Seq database with the accession number GSE37604. Asterisks indicate motifs with CAXXTG sequences.

No.	Motif	*P* value	No.	Motif	*P* value
1*	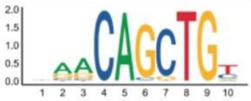	1×10^{-37}	9*	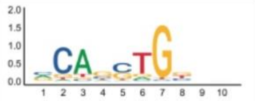	1×10^{-20}
2	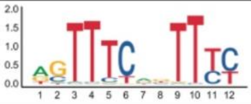	1×10^{-33}	10	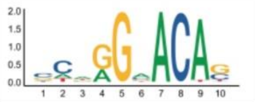	1×10^{-19}
3*	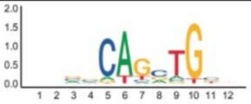	1×10^{-26}	11	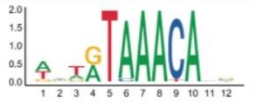	1×10^{-19}
4	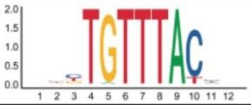	1×10^{-26}	12	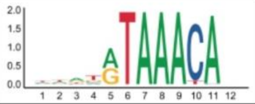	1×10^{-16}
5*	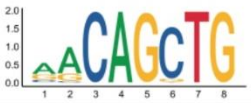	1×10^{-25}	13	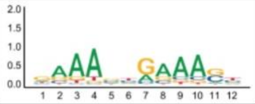	1×10^{-16}
6*	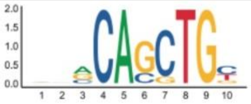	1×10^{-21}	14	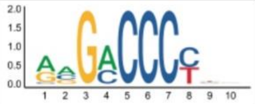	1×10^{-15}
7	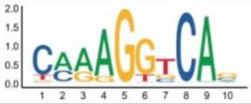	1×10^{-21}	15*	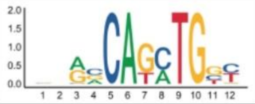	1×10^{-14}
8	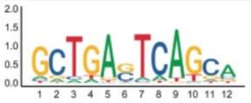	1×10^{-20}	16*	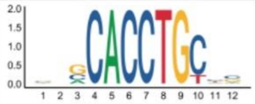	1×10^{-13}
